# The cys-loop ligand-gated ion channel gene superfamily of the red flour beetle, *Tribolium castaneum*

**DOI:** 10.1186/1471-2164-8-327

**Published:** 2007-09-19

**Authors:** Andrew K Jones, David B Sattelle

**Affiliations:** 1 MRC Functional Genetics Unit, Department of Physiology Anatomy and Genetics, The Sherrington Building, University of Oxford, South Parks Road, Oxford, OX1 3QX, UK

## Abstract

**Background:**

Members of the cys-loop ligand-gated ion channel (cys-loop LGIC) superfamily mediate chemical neurotransmission and are studied extensively as potential targets of drugs used to treat neurological disorders such as Alzheimer's disease. Insect cys-loop LGICs are also of interest as they are targets of highly successful insecticides. The red flour beetle, *Tribolium castaneum*, is a major pest of stored agricultural products and is also an important model organism for studying development.

**Results:**

As part of the *T. castaneum *genome sequencing effort, we have characterized the beetle cys-loop LGIC superfamily which is the third insect superfamily to be described after those of *Drosophila melanogaster *and *Apis mellifera*, and also the largest consisting of 24 genes. As with *Drosophila *and *Apis*, *Tribolium *possesses ion channels gated by acetylcholine, γ-amino butyric acid (GABA), glutamate and histamine as well as orthologs of the *Drosophila *pH-sensitive chloride channel subunit (pHCl), CG8916 and CG12344. Similar to *Drosophila *and *Apis*, *Tribolium *cys-loop LGIC diversity is broadened by alternative splicing although the beetle orthologs of RDL and GluCl possess more variants of exon 3. Also, RNA A-to-I editing was observed in two *Tribolium *nicotinic acetylcholine receptor subunits, Tcasα6 and Tcasβ1. Editing in Tcasα6 is evolutionarily conserved with *D. melanogaster*, *A. mellifera *and *Heliothis virescens*, whereas Tcasβ1 is edited at a site so far only observed in the beetle.

**Conclusion:**

Our findings reveal that in diverse insect species the cys-loop LGIC superfamily has remained compact with only minor changes in gene numbers. However, alternative splicing, RNA editing and the presence of divergent subunits broadens the cys-loop LGIC proteome and generates species-specific receptor isoforms. These findings on *Tribolium castaneum *enhance our understanding of cys-loop LGIC functional genomics and provide a useful basis for the development of improved insecticides that target an important agricultural pest.

## Background

In insects, members of the cys-loop ligand-gated ion channel (cys-loop LGIC) superfamily mediate both fast excitatory and inhibitory synaptic transmission in the nervous system. The superfamily includes cation permeable nicotinic acetylcholine receptors (nAChRs) [1,2], γ-amino butyric acid (GABA)-gated anion channels [3], glutamate-gated chloride channels (GluCls) [4] and histamine-gated chloride channels (HisCls) [5,6]. Studies of *Drosophila melanogaster *and *Apis mellifera *have shown that cys-loop LGICs mediate important aspects of behaviour such as escape response [7], learning and memory [8-12]. Members of the cys-loop superfamily of ionotropic receptors are also of considerable interest as they are targets of widely used insecticides [13]. For example, nAChRs are targets of neonicotinoids [14,15], a class of insect control chemicals which include imidacloprid with worldwide annual sales of approximately one billion US dollars [16]. Also, GABA receptors, GluCls and HisCls are targets of fipronil and avermectins [17,18].

The red flour beetle, *Tribolium castaneum*, is a highly sophisticated genetic and developmental model organism [19], and is a major global pest of stored agricultural products. In the USA alone, *T. castaneum *contributes to over one billion US dollars worth of damage to wheat and corn every year [20]. *Tribolium *has proven highly adaptable, developing resistance to a wide range of insecticides raising the need for the development of improved and novel control agents. To date, partial DNA sequences of only a few *T. castaneum *cys-loop LGIC subunits have been reported. These include a HisCl [21], transcript variants of a nAChR subunit orthologous to *Drosophila melanogaster *Dα6 (submitted to NCBI by Jin and colleagues, Acession Numbers EF127806–EF127810) and a GABA-gated ion channel which is an ortholog of *Drosophila *RDL [22]. Interestingly, a mutation changing alanine 302 to serine in the *Tribolium *GABA receptor is associated with cyclodiene resistance [23,24]. Indeed, the same mutation is found in RDL from cyclodiene resistant strains of a wide range of insect species [25,26]. Characterizing the full complement of *Tribolium *cys-loop LGIC subunits represents a critical step in identifying key components of the beetle nervous system as well pinpointing particular insecticide targets. Here we have used sequence data from the *T. castaneum *genome project [27] to provide the first description of a complete cys-loop LGIC gene superfamily from an invertebrate pest species.

## Results

### The *T. castaneum *cys-loop LGIC superfamily consists of 24 subunit members

**Figure 1:**
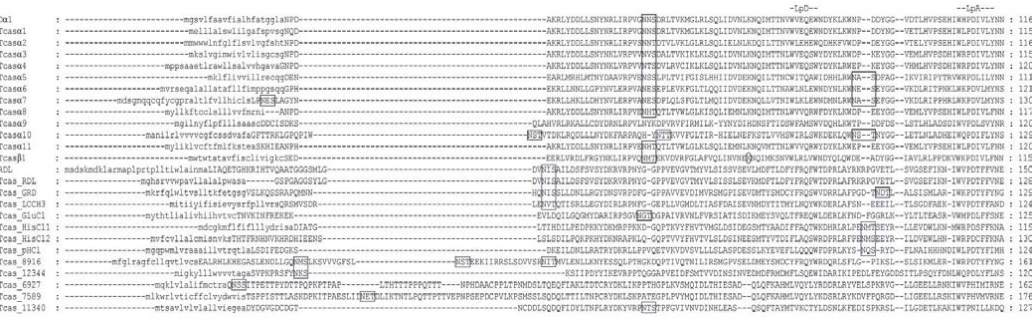
This figure shows the upper quartile of a protein sequence alignment of *T. castaneum *cys-loop LGIC subunits, for the full image please see Additional file 2. *Drosophila *Dα1 and RDL are included for comparison. N-terminal signal leader peptides are shown in lower case and loops implicated in ligand binding (LpA-F) are indicated. Putative N-glycosylation sites are boxed and amino acid residues altered by RNA editing are circled.

**Figure 2:**
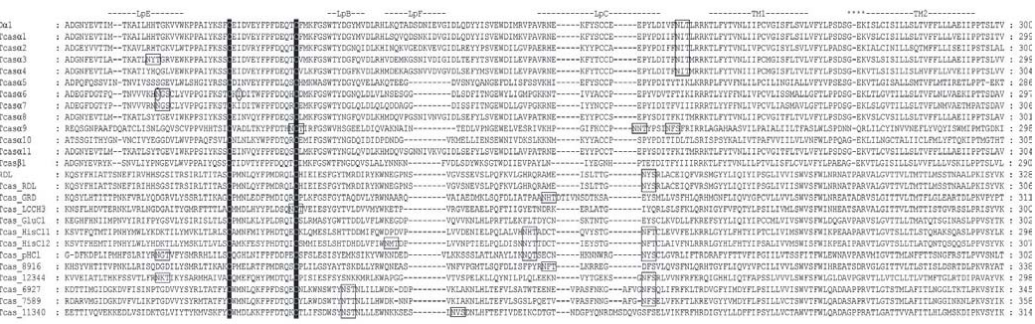
This figure shows the second quartile of a protein sequence alignment of *T. castaneum *cys-loop LGIC subunits, for the full image please see Additional file 2. *Drosophila *Dα1 and RDL are included for comparison. Loops implicated in ligand binding (LpA-F) as well as transmembrane regions (TM) are indicated. The two cysteines forming the cys-loop are highlighted in black shading. Putative N-glycosylation sites are boxed and amino acid residues altered by RNA editing are circled.

**Figure 3:**
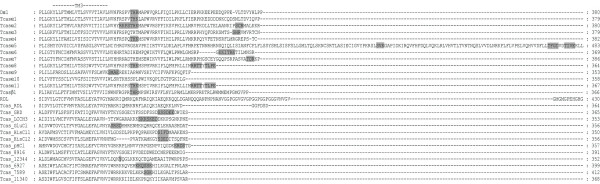
This figure shows the third quartile of a protein sequence alignment of *T. castaneum *cys-loop LGIC subunits, for the full image please see Additional file 2. *Drosophila *Dα1 and RDL are included for comparison. Transmembrane regions (TM) are indicated and potential cAMP, PKC and CK2 phosphorylation sites are boxed with gray shading while potential tyrosine kinase phosphorylation sites are enclosed in gray shaded ovals.

**Figure 4:**
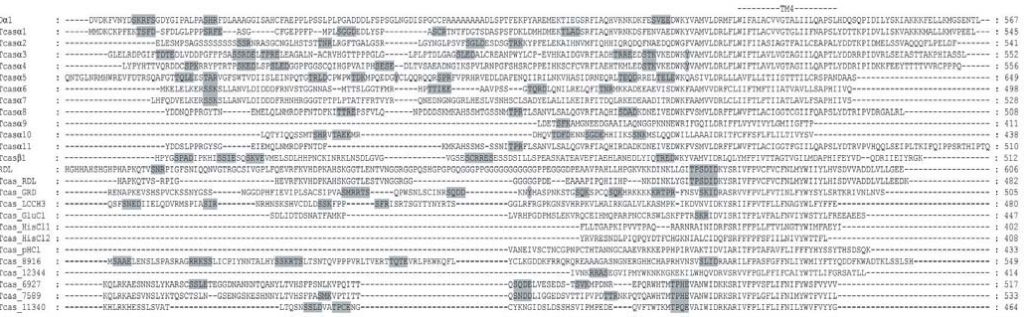
This figure shows the lower quartile of a protein sequence alignment of *T. castaneum *cys-loop LGIC subunits, for the full image please see Additional file 2. *Drosophila *Dα1 and RDL are included for comparison. Transmembrane regions (TM) are indicated and potential cAMP, PKC and CK2 phosphorylation sites are boxed with gray shading while potential tyrosine kinase phosphorylation sites are enclosed in gray shaded ovals.

Using tBLASTn [28], 24 candidate cys-loop LGICs were identified in the *T. castaneum *genome and manually annotated. This is the third complete insect cys-loop LGIC superfamily to be described after those of *D. melanogaster *and *A. mellifera *and the largest known to date since the fruit fly possesses 23 subunits and the honey bee has 21 [29]. RT-PCR [see Additional file 1 for primers used] showed that all of the *Tribolium *cys-loop LGIC subunits are transcribed. An alignment of their protein sequences shows that the beetle subunits possess features common to members of the cys-loop LGIC superfamily [30] (Figs. 1, 2, 3, 4 and Additional file 2). These include: (a) an extracellular N-terminal domain containing distinct regions (loops A-F) that form the ligand binding site [31]; (b) the dicysteine-loop (cys-loop) consisting of two disulphide bond-forming cysteines separated by 13 amino acid residues; (c) four transmembrane regions (TM1–4), the second of which (TM2) contributes most of the channel lining residues; (d) a highly variable intracellular loop between TM3 and TM4. As with other cys-loop LGIC subunits, the *Tribolium *sequences also possess potential N-glycosylation sites in the extracellular N-terminal domain and phosphorylation sites in the TM3–TM4 intracellular loop.

**Figure 5:**
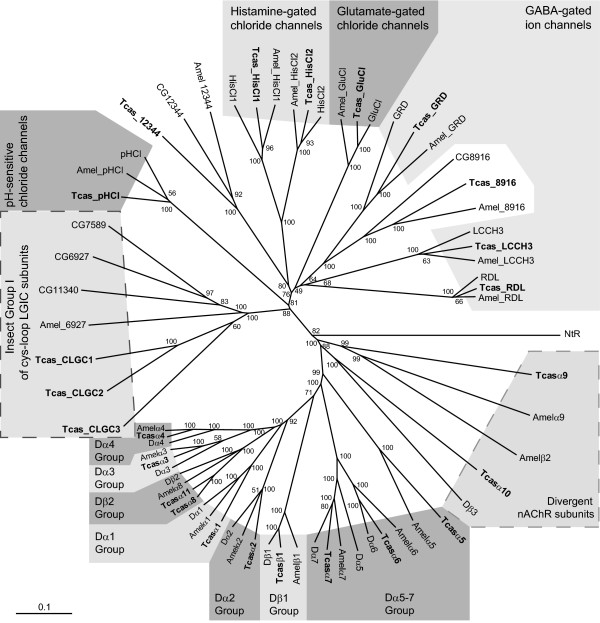
Tree showing relationships of *T. castaneum*, *A. mellifera *and *D. melanogaster *cys-loop LGIC subunit protein sequences. Numbers at each node signify bootstrap values with 100 replicates and the scale bar represents substitutions per site. The subunits shown in the tree are as follows: *A. mellifera *Amelα1 (DQ026031), Amelα2 (AY540846), Amelα3 (DQ026032), Amelα4 (DQ026033), Amelα5 (AY569781), Amelα6 (DQ026035), Amelα7 (AY500239), Amelα8 (AF514804), Amelα9 (DQ026037), Amelβ1 (DQ026038), Amelβ2 (DQ026039), Amel_GluCl (DQ667185), Amel_GRD (DQ667183), Amel_HisCl1 (DQ667187), Amel_HisCl2 (DQ667188), Amel_LCCH3 (DQ667184), Amel_pHCl (DQ667189), Amel_RDL (DQ667182), Amel_6927 (DQ667195), Amel_8916 (DQ667193), Amel_12344 (DQ667194); *D. melanogaster *Dα1 (CAA30172), Dα2 (CAA36517), Dα3 (CAA75688), Dα4 (CAB77445), Dα5 (AAM13390), Dα6 (AAM13392), Dα7 (AAK67257), Dβ1 (CAA27641), Dβ2 (CAA39211), Dβ3 (CAC48166), GluCl (AAG40735), GRD (Q24352), HisCl1 (AAL74413), HisCl2 (AAL74414), LCCH3 (AAB27090), Ntr (AF045471), pHCl (NP_001034025), RDL (AAA28556), CG6927 (AAF45992), CG7589 (AAF49337), CG8916 (BT022901), CG11340 (AAF57144), CG12344 (AAF58743); *T. castaneum *subunits, which are shown in boldface type, Tcasα1 (EF526080), Tcasα2 (EF526081), Tcasα3 (EF526082), Tcasα4 (EF526083), Tcasα5 (EF526085), Tcasα6 (EF526086), Tcasα7 (EF526089), Tcasα8 (EF526090), Tcasα9 (EF526091), Tcasα10 (EF526092), Tcasα11 (EF526093), Tcasβ1 (EF526094), Tcas_CLGC1 (EF545129), Tcas_CLGC2 (EF545130), Tcas_CLGC3 (EF545131), Tcas_GluCl (EF545121), Tcas_GRD (EF545119), Tcas_HisCl1 (EF545124), Tcas_HisCl2 (EF545125), Tcas_LCCH3 (EF545120), Tcas_pHCl (EF545126), Tcas_RDL (EF545117), Tcas_8916 (EF545127), Tcas_12344 (EF545128).

A comparison of sequence identities between *T. castaneum, D. melanogaster *and *A. mellifera *cys-loop LGIC subunits (Tables 1 and 2), as well as the use of a phylogenetic tree (Fig. 5), indicates orthologous relationships between the beetle, honey bee and fruit fly subunits. To facilitate comparisons between the three species, *Tribolium *subunits were named after their *Drosophila *counterparts as previously done with *Apis *subunits [29]. For example, the beetle orthologs of *Drosophila *Dα1, RDL and CG8916 were designated Tcasα1, Tcas_RDL and Tcas_8916 respectively.

**Table 1:** Percentage identity/similarity between *T. castaneum *and *A. mellifera *nAChR subunit protein sequences. Proposed orthologs are shown in bold.

Subunit	Tcasα1	Tcasα2	Tcasα3	Tcasα4	Tcasα5	Tcasα6	Tcasα7	Tcasα8	Tcasβ1	Tcasα9	Tcasα10	Tcasα11
Linkage Group	2	9	chrUn_77	2	4	5	chrUn_37	1	chrUn_37	7	4	1
Amelα1	**68/75**	46/62	53/64	49/62	22/40	31/44	30/44	48/59	36/51	14/30	17/33	46/59
Amel α2	53/67	**78/84**	49/65	46/62	21/38	33/51	34/52	50/65	38/55	16/35	21/37	50/66
Amel α3	54/66	48/62	**84/89**	65/74	22/38	32/48	32/49	54/67	38/53	15/32	18/35	53/65
Amel α4	52/63	46/61	67/77	**87/90**	23/39	33/50	32/49	51/66	38/54	15/32	18/35	51/65
Amel α5	30/46	27/47	29/45	27/44	**48/59**	32/51	30/48	29/47	30/49	17/35	20/40	28/45
Amel α6	31/48	33/52	32/49	32/48	25/40	**73/83**	63/74	33/52	33/52	15/36	21/39	33/49
Amel α7	30/47	33/49	32/47	31/46	25/42	58/69	**67/75**	31/48	31/50	14/31	19/36	30/47
Amel α8	54/67	49/66	56/68	52/67	21/36	35/52	34/51	**70/80**	40/56	15/35	21/37	69/79
Amelβ1	38/54	38/55	39/56	38/54	22/38	34/54	32/51	39/57	**83/90**	15/34	20/37	38/56
Amel β2	11/30	12/31	11/29	10/30	10/24	13/33	14/32	12/32	13/34	23/35	16/38	12/32
Amel α9	14/31	14/32	14/31	15/32	12/25	15/34	14/32	16/34	14/32	28/51	22/41	15/35

**Table 2:** Percentage identity/similarity between *T. castaneum *and *D. melanogaster *non-nAChR subunit protein sequences. Proposed orthologs are shown in bold.

Subunit	Tcas_RDL	Tcas_GRD	Tcas_LCCH3	Tcas_GluCl	Tcas_HisCl1	Tcas_HisCl2	Tcas_pHCl	Tcas_8916	Tcas_12344	CLGC1	CLGC2	CLGC3
Linkage Group	chrUn_22	4	4	chrUn_11	4	1	2	4	1	8	8	8
RDL	**69/73**	24/37	27/43	22/34	19/32	20/33	17/31	21/35	19/31	17/31	16/30	15/30
GRD	25/38	**54/62**	24/38	21/34	18/33	18/32	15/26	34/48	17/30	17/29	17/30	14/30
LCCH3	31/48	27/43	**73/82**	25/42	23/40	25/39	17/34	24/38	20/36	18/36	17/36	17/34
GluCl	27/41	22/39	26/42	**83/89**	27/42	27/45	24/44	22/38	21/39	19/36	19/35	19/37
HisCl1	21/38	20/38	25/41	26/42	**64/70**	43/57	19/37	19/34	22/42	18/35	18/34	16/35
HisCl2	24/41	21/37	25/40	26/43	50/64	**79/85**	21/39	20/35	26/46	19/37	18/37	18/35
pHCl	18/33	15/28	17/32	23/40	19/36	19/36	**68/74**	15/27	17/34	21/36	18/31	19/35
CG8916	22/37	32/47	23/38	19/34	19/30	19/33	15/28	**54/66**	17/29	15/30	15/30	15/28
CG12344	22/39	21/35	20/36	21/39	25/44	25/45	19/36	16/30	**54/71**	18/33	17/32	18/35
CG6927	19/35	17/31	18/37	18/34	17/33	17/36	19/33	15/30	16/32	38/54	36/51	30/48
CG7589	18/35	16/31	20/39	19/36	17/34	18/35	18/31	16/34	18/34	37/56	36/52	32/51
CG11340	19/34	15/29	17/36	20/35	17/35	18/35	19/34	16/32	17/32	32/50	34/50	27/45
Ntr	8/21	9/23	10/23	9/25	10/24	11/25	11/26	8/20	8/23	8/23	8/21	9/28

### *Tribolium *nicotinic acetylcholine receptor subunits

We identified 12 candidate nAChR subunit-encoding genes in the *T. castaneum *genome. This is the largest insect nAChR gene family so far described as those of *D. melanogaster*, *Anopheles gambiae *and *A. mellifera *consist of 10, 10 and 11 subunits respectively [1,32,33]. Eleven of the *Tribolium *nAChR subunits possess the two adjacent cysteine residues in loop C (Fig. 2) which are important for acetylcholine (ACh) binding [34], defining them as α subunits. The remaining subunit was designated β since it lacks the vicinal cysteines.

*Tribolium *possesses core groups of nAChR subunits that are highly conserved between different insect species [35]. Thus, subunit equivalents of Dα1–7, Dβ1 and Dβ2 are evident in the beetle genome (Fig. 5). As with *Anopheles*, *Apis *and several other insects [36], the *Tribolium *ortholog of Dβ2 is of the α type (Tcasα8). Interestingly, the beetle possesses an additional α subunit (Tcasα11) in the Dβ2 group, indicating a gene duplication in the *Tribolium *lineage. In line with this, both Tcasα8 and Tcasα11 genes are tightly clustered together within 8 kb of each other in the beetle genome suggesting that both subunits arose from a recent duplication event from a common gene. Both *A. gambiae *and *D. melanogaster *possess three subunits (Dα5, Dα6 and Dα7) that are very similar and show substantial homology to the vertebrate α7 nAChR subunit [32,37]. In *T. castaneum*, two orthologs of these subunits (Tcasα6 and Tcasα7) are also similar to human α7, sharing 46% and 45% identity respectively at the protein level. The third subunit, Tcasα5, when compared with vertebrate nAChR subunits, is most similar to α7 but shares only 25% identity and, along with its *Apis *ortholog (Amelα5), departs strongly from Dα5 (Fig. 5). *Tribolium *nAChR subunits outside of the Dα5–7 group show 19%–39% identity with vertebrate subunits. As is the case for Dα1, Dα2, Dα3, Dα4, Dβ2 and their *Anopheles *and *Apis *orthologs, the corresponding *Tribolium *subunits (Tcasα1–α4, Tcasα8 and Tcasα11) have an insertion in loop F (Fig. 2), which may contribute to imidacloprid interactions [38]. The Dα1, Dα2 and Dβ2 genes, as well as their *Anopheles *orthologs, Agamα1, Agamα2 and Agamα8, are similarly arranged and tightly clustered within 200 kb and 220 kb respectively [32]. Immunohistochemical and coimmunoprecipitation studies show that Dα1, Dα2 and Dβ2 are integral components of certain nAChRs subtypes, leading to the suggestion that clustering may facilitate coordinated expression and co-assembly of the nAChR subunits [39]. In *Apis*, Amelα1 and Amelα2 are clustered but are separated from the honey bee Dβ2 ortholog, Amelα8 [33], while in the *Tribolium *genome all equivalent beetle subunits, Tcasα1, Tcasα2 and Tcasα8, are located on different linkage groups (Table 1). The separation of these genes may thus result in diversification of receptor expression and coassembly. *Tribolium *does however, show clustering of Tcasα7 and Tcasβ1 (both genes lie within 8 kb of each other) which is conserved in the genomes of *Anopheles *and *Apis *but not *Drosophila *[33].

Analysis of *D. melanogaster*, *A. gambiae *and *A. mellifera *nAChR gene families has shown that each insect possesses at least one divergent subunit that shares relatively low sequence identity with other nAChR subunits [35]. The four insect nAChR gene families described so far each contain a different complement of divergent subunits. Thus, *Drosophila *and *Anopheles *have one divergent subunit each but are of the β and α types respectively [32,40], while *Apis *and *Tribolium *each have two subunits which are α and β in the honey bee and are both α subunits in the beetle [33] (Fig. 5). One of the *Tribolium *divergent subunits, Tcasα9, possesses an atypical FxCC amino acid motif instead of YxCC found in loop C of all other insect α nAChR subunits characterised to date (Fig. 2). The nematode, *Caenorhabditis elegans*, is the only other organism known to possess nAChR subunits with the FxCC motif [41]. Since site-directed mutagenesis has shown that a Tyr to Phe substitution in the heterologously expressed vertebrate α7 subunit results in a tenfold lower affinity for ACh [42], Tcasα9 may have unusual ligand binding properties. In addition, Tcasα9 lacks the GEK motif characteristic of nAChR subunits which precedes TM2 (Fig. 2) and plays an important role in ion permeation and selectivity [43]. Most notably, the absence of the highly conserved glutamic acid residue may have given rise to a receptor with distinct ion channel properties since a substitution of the equivalent glutamic acid residue in the vertebrate α7 nAChR abolishes permeability to calcium ions but not monovalent cations [44].

**Figure 6:**
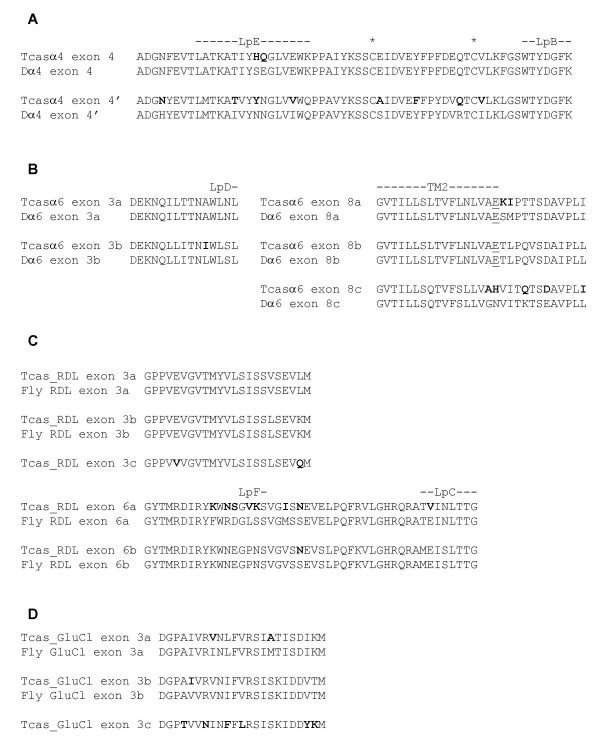
Alternative splicing of exons in *T. castaneum *cys-loop LGIC subunits. Equivalent alternate exons of *T. castaneum *and *D. melanogaster *cys-loop LGIC subunits are aligned. (A) Exon 4 splice variants in Tcasα4 and Dα4. The cysteine residues forming the cys-loop are marked by asterisks. (B) Splice variants of exons 3 and 8 in both Tcasα6 and Dα6. The glutamic acid residue located in the second transmembrane region (indicated as TM2) and involved in ion conductance [48] is underlined. (C) Splice variants of exons 3 and 6 in both Tcas_RDL and *Drosophila *RDL. Tribolium has an additional alternative for exon 3 (denoted Tcas_RDL exon 3c). (D) Exon 3 splice variants in Tcas_GluCl and *Drosophila *GluCl. Tribolium has an additional alternative exon (denoted Tcas_GluCl exon 3c). Throughout the figure, *Tribolium *residues that differ from those of the orthologous *Drosophila *exon are highlighted in bold and loops B to F, which contribute to ligand binding, are indicated.

Two *Tribolium *nAChR subunits, Tcasα4 and Tcasα6, have alternatively spliced exons most likely arising from tandem exon duplication [45]. This alternative splicing is conserved in *Anopheles*, *Apis *and *Drosophila*. Thus, as with Agamα4, Amelα4 and Dα4 [32,33,46], Tcasα4 possesses two alternatives for exon 4 (denoted exon4 and exon4') (Fig. 6A), and similar to Dα6 [37], Tcasα6 has two alternatives for exon 3 and three alternatives for exon 8 (Fig. 6B). Analysis of sequence chromatograms shows that both alternatives for Tcasα4 exon4 are transcribed while RT-PCR [see Additional file 3 for primers used] detected all six possible combinations of alternate exons for Tcasα6. As previously observed for *Anopheles*, *Apis *and *Drosophila *nAChRs, alternative splicing introduces amino acid changes in functionally significant regions and thus is likely to increase nAChR diversity [32,33,37,46]. Thus, alternative splicing of Tcasα4 exon4 substitutes residues in loop E, which may affect ligand binding [31], as well as residues in the vicinity of the cys-loop which may affect receptor assembly [46,47]. For Amelα6, alternative splicing of exon 8 changes residues in the TM2 domain which may alter the ion channel properties of the receptor. In one example, Tcasα6 exon 8c substitutes a highly conserved glutamic acid residue (Fig. 6B), which may affect ion conductance [48]. Two *Tribolium *alternate exons, Tcasα6 exon 3a and Tcasα6 exon 8b, have sequences that are identical to the equivalent exons in *Drosophila *(Fig. 6B). Also, Tcasα6 exon 8b is completely conserved in *Anopheles *and *Apis*, indicating an evolutionarily robust function for this exon. As highlighted in Fig. 6, the other alternate exons of Tcasα4 and Tcasα6 have residues that differ from those of their *Drosophila *counterparts which may give rise to nAChR splice variants with functional properties particular to certain insect species.

**Figure 7:**
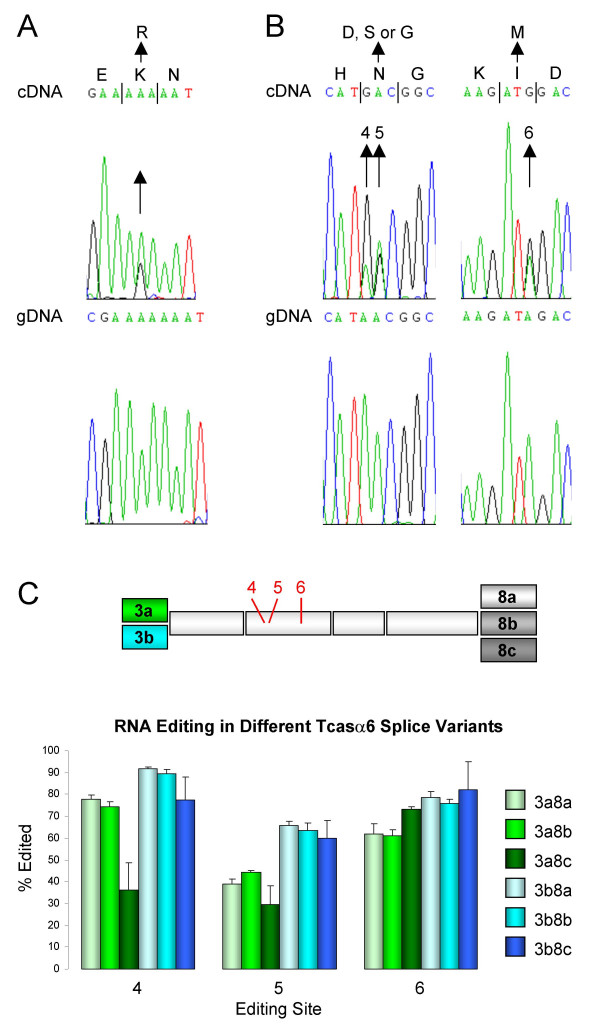
RNA A-to-I editing in *T. castaneum *cys-loop LGIC subunits. Arrows highlight the mixed adenosine/guanosine peak in the cDNA sequence indicating RNA editing as well as the resulting amino acid change. The corresponding genomic DNA (gDNA) sequence, which lacks this mixed A/G signal, is also shown. (A) RNA editing of Tcasβ1. (B) RNA editing of Tcasα6. Editing sites 4, 5 and 6 [37] are indicated. (C) A schematic of exons 3–8 of Tcasα6 with editing sites 4–6 (indicated in red) is shown. The graph shows mean RNA editing levels (n = 4) at sites 4–6 in different splice variants. Error bars indicate standard deviation.

Five *D. melanogaster *nAChR subunits (Dα5, Dα6, Dα7, Dβ1 and Dβ2) as well as RDL and GluCl are known to undergo pre-mRNA A-to-I editing [7,37,49,50], a process which essentially converts adenosine (A) in the genome into guanosine (G) in transcripts, thereby generating mRNA with a nucleotide sequence that differs from the corresponding genomic DNA [51]. The cDNA sequences of all the *Tribolium *cys-loop LGIC subunits were analyzed and potential RNA editing was observed in two nAChR subunits, Tcasα6 and Tcasβ1 (Fig. 7). Sequencing of the corresponding genomic DNA verified that the nucleotide changes occur at the RNA level. Tcasβ1 is edited at a single site which alters a highly conserved lysine to an arginine residue in the vicinity of loop D, potentially affecting the subunit's ligand binding properties (Fig. 1 and Fig. 7A). Editing at this site has not been observed for any other nAChR subunits although residues nearby are edited in Dβ1 [49]. Tcasα6, on the other hand, demonstrates a high degree of evolutionary conservation in RNA editing as it undergoes editing that alters two amino acid residues as is also the case for the orthologs of *D. melanogaster*, *A. mellifera *and the tobacco budworm, *Heliothis virescens *[33,37]. Two of the editing sites in Tcasα6 (Fig. 7B), corresponding to sites 4 and 5 in Dα6 [37], remove a potential N-glycosylation site which may affect receptor maturation, channel desensitization and conductance [52,53]. We analyzed the RNA editing levels at sites 4–6 in RT-PCR products generated by primers that amplify specific splice variants [see Additional file 3 for primers used]. As shown in Fig. 7C, the extent of editing varies between the three sites (two-way ANOVA analysis P < 0.0001). For instance, editing at site 4 in the exon3b+exon8a (3b8a), 3b8b and 3b8c splice variants is significantly higher than site 5 editing of the same isoforms (P < 0.001). More strikingly, RNA editing levels at the same site vary with the splice variant. For example, the 3a8c isoform has notably low editing levels at site 4 when compared to the other splice variants (one-way ANOVA P < 0.001). In another case, the minority of transcripts with exon 3a is edited at site 5 while the majority of isoforms with exon 3b are edited at the same site (P < 0.01). This suggests that RNA-editing and alternative splicing are linked in generating proteome diversity. This is in accord with findings of a study investigating the relationship between the two processes in the *Drosophila *Dα5 nAChR subunit [54].

### *Tribolium *GABA-gated ion channels

The *Tribolium *genome contains orthologs of the three known *D. melanogaster *GABA-gated ion channels, RDL, GRD and LCCH3 [3] (Fig. 5 and Table 2). RDL possesses a PAR sequence before TM2 which is characteristic of cys-loop ligand-gated anion channels [43] while GRD and LCCH3 lack this sequence. This may underlie the findings that RDL forms homomeric anion channels [26] whereas GRD and LCCH3 form heteromultimeric cation channels when expressed in *Xenopus laevis *oocytes [55]. The PAR motif is also present in Tcas_RDL and is absent in Tcas_GRD and Tcas_LCCH3 (Fig. 2) indicating that *Tribolium *may possess both ligand-gated anion and cation channels. However, whereas electrophysiology has clearly shown that GABA induces inhibitory chloride channels in insects, it remains to be established whether GABA-gated cation channels function *in vivo *[3].

As is the case for *Drosophila *RDL, exons 3 and 6 are alternatively spliced in Tcas_RDL (Fig. 6C) although, whereas *Drosophila *and *Apis *have two alternatives for exon 3 [29,56], *Tribolium *has three. To accommodate this extra alternative exon and anticipate the possibility of additional exons in other insect species, we have revised the nomenclature of RDL splice variants. Thus, RDL exons "a" and "b" are denoted exon 3a and exon 3b respectively, while exons "c" and "d" are now called exon 6a and exon 6b. We have designated the extra exon in Tcas_RDL as exon 3c. Exons 3a and 3b are completely conserved in *Drosophila *RDL [56], Amel_RDL [29] and Tcas_RDL, while exon 3c of *Tribolium *introduces two novel amino acid residues near to loop D (Fig. 6C). RT-PCR [see Additional file 3 for primers used] show that all six possible combinations of alternate exons for Tcas_RDL are transcribed. Since studies of *Drosophila *RDL demonstrate that alternative splicing alters affinity for GABA [57], the extra choice of exon in Tcas_RDL may give rise to a receptor with extended functional range when compared to orthologs in other insects. The most variation seen between a Tcas_RDL alternate exon and its equivalent in *Drosophila *is in exon 6a which differ by eight residues, most of which are located in the vicinity of loop F (Fig. 6C). This may give rise to receptor variants that have distinct ligand binding characteristics in the two insect species.

**Figure 8:**
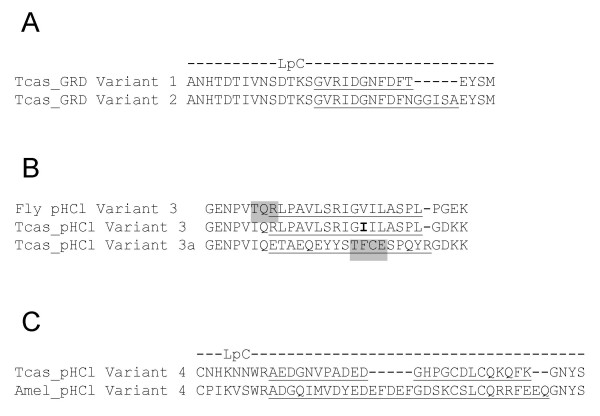
Differential splicing in *T. castaneum *cys-loop LGIC subunits. (A) Alignment of loop C (LpC) sequences of Tcas_GRD variants 1 and 2. Insertions arising from differential use of splice sites are underlined. (B) Alignment of variant 3 of *Drosophila *pHCl with the equivalent *Tribolium *variant (Tcas_pHCl Variant 3). The variants are caused by the differential use of splice sites which inserts stretches of amino acids (underlined). The *Tribolium *residue in Tcas_pHCl Variant 3 that differs from that of the equivalent *Drosophila *splice variant is highlighted in bold. *Tribolium *has an additional variant, Tcas_pHCl Variant 3a, resulting from an insertion of a different peptide sequence at the same site. Potential phosphorylation sites are highlighted in gray shading. (C) Alignment of loop C (LpC) sequences of Tcas_pHCl Variant 4 and a similar variant in *Apis *(Amel_pHCl Variant 4) where use of differential splice sites introduces an insertion (underlined).

Variants of Tcas_GRD were detected in RT-PCR products where differential use of splice sites introduces either one of two insertions in loop C which are denoted variant 1 or variant 2 (Fig. 8A). Variations of *Drosophila *or *Apis *GRD subunits have not so far been observed, although it has been noted that the fruit fly GRD subunit has an unusual stretch of 75 amino acids which is present at the site equivalent to Tcas_GRD variants 1 and 2 [58]. Since loop C is involved in ligand binding [31], differential splicing has the potential to diversify the ligand binding properties of Tcas_GRD.

### *Tribolium *glutamate and histamine-gated chloride channels

As with *D. melanogaster *and *A. mellifera*, *T. castaneum *has one known glutamate-gated chloride channel (Tcas_GluCl) and two histamine-gated chloride channels (Tcas_HisCl1 and Tcas_HisCl2). Consistent with their putative function as anion channels, Tcas_GluCl, Tcas_HisCl1 and Tcas_HisCl2 all have the PAR motif preceding TM2 (Fig. 2) [43]. Out of the ligand-gated anion channels, GluCl and HisCl2 are the most highly conserved between the fruit fly and beetle, sharing 83% and 79% identity respectively (Table 2).

Exon 3 of Tcas_GluCl is alternatively spliced as is the case with *Drosophila *GluCl and *Apis *GluCl [33,50]. However, whereas the fruit fly and honey bee GluCls each have two alternatives for exon 3, *Tribolium *has three (Fig. 6D). Sequence chromatograms of Tcas_GluCl RT-PCR products [using primers shown in Additional file 1] showed mixed peaks corresponding to exon 3 indicating multiple variants are transcribed. In order to maintain consistency with other cys-loop LGIC subunits, we have revised the nomenclature of GluCl alternative exons so that Modules 1 and 2 correspond to exons 3a and 3b respectively. Similar to RDL, alternative splicing of GluCl alters amino acid residues in the vicinity of loop D which may introduce variation in ligand-binding properties. With the extra alternative exon (exon 3c), Tcas_GluCl may have a wider range in receptor functional properties when compared to GluCls of other insect species.

### *Tribolium *pHCl and uncharacterized cys-loop LGIC subunits

The pH sensitive chloride channel (pHCl) first identified in *Drosophila *[59] is well conserved in *T. castaneum*, showing 68% identity (Table 2). The *Drosophila *pHCl has several splice variants, of which Variant 3 introduces an insertion in the intracellular region between TM3 and TM4. We detected a similar variant in Tcas_pHCl transcripts (Fig. 8B) where the peptide insertion differs by only one amino acid residue from that of *Drosophila *pHCl [59] and is completely identical to the equivalent insertion in Amel_pHCl [29]. However, unlike *Drosophila *and *Apis*, the Variant 3 insertion does not introduce a potential protein kinase C phosphorylation site in Tcas_pHCl. This may lead to the beetle pHCl having distinct characteristics since phosphorylation of the large intracellular region modulates receptor assembly and function [60,61], Interestingly, we detected a second insertion at the same site which has not been observed in *Drosophila *or *Apis*. Denoted Tcas_pHCl Variant 3a (Fig. 8B), this insertion introduces a putative casein kinase II phosphorylation site. Tcas_pHCl also has an insertion in loop C (Variant 4, Fig. 8C) which is likely to impact on ligand binding. The equivalent of this insertion has not been observed in *Drosophila *but has been detected in *Apis *with the insertion sequence and length differing considerably between beetle and honey bee (Fig. 8C).

Five *Drosophila *cys-loop LGIC subunits have yet to be functionally characterized. These are CG6927, CG7589, CG8916, CG11340 and CG12344. CG8916 and CG12344 appear to be closely related with GRD and HisCls respectively while CG6927, CG7589 and CG11340 forms a distinct subfamily of cys-loop LGIC subunits which, following on from a recent study [62], we have denoted Insect Group 1 of cys-loop LGIC subunits (Fig. 5). *Tribolium *also possesses five uncharacterized subunits. Two of these, Tcas_8916 and Tcas_12344, are candidate orthologs of CG8916 and CG12344 being notably similar to their *Drosophila *counterparts, both sharing 54% identity (Table 2). The remaining three subunits belong to Insect Group I (Fig. 5) and have been denoted Tcas_CLGC1, Tcas_CLGC2 and Tcas_CLGC3, standing for *c*ys-loop *l*igand *g*ated ion *c*hannel. Despite sharing highest sequence identities with *Drosophila *Insect Group I subunits (Table 2), their orthologous relationships are uncertain since, for example, Tcas_CLGC2 shows 36% identity with both CG6927 and CG7589 (Table 2). *A. mellifera *possesses only one subunit, Amel_6927, in Insect Group 1 [29], indicating that gene duplication occurred after the emergence of the Hymenoptera to give rise to the three subunits present in both *Tribolium *(Coleoptera) and *Drosophila *(Diptera). This is in line with recent findings suggesting that the Hymenoptera are basal to the Coleoptera [63,64]. Consistent with gene duplication occurring within Insect Group 1 just before the emergence of Coleoptera, Tcas_CLGC1, Tcas_CLGC2 and Tcas_CLGC3 are tightly clustered together in the *Tribolium *genome, being located within 10 Kb in linkage group 8. In the more evolutionarily advanced *D. melanogaster *[63], the three genes are separated with CG7589 and CG11340 being respectively located on the left and right arms of chromosome 3 and CG6927 being present on chromosome X [65].

## Discussion

Insect genome sequencing projects have allowed the identification and comparison of gene superfamilies from diverse species. As part of the *Tribolium *sequencing project [27], we have described the beetle cys-loop LGIC superfamily which encodes for receptors that play major roles in the nervous system and are also targets of highly successful insecticides. This is the first complete cys-loop LGIC superfamily to be described from a Coleoptera and an agricultural pest species, and is the third to be reported after those of the Dipteran *D. melanogaster *[29,62,66] and the Hymenopteran *A. mellifera *[29]. In the three insect species, which represents over 300 million years of evolution [63], the cys-loop LGIC superfamily has remained compact with only minor changes in gene numbers. However, alternative splicing and RNA A-to-I editing have considerably increased receptor diversity, effectively introducing changes in functionally significant and highly conserved regions to generate subunit isoforms particular to certain insect species. Also, it is becoming apparent that each insect possesses a distinct complement of highly divergent nAChR subunits whose sequences do not reflect a high degree of evolutionary constraint and thus may play diverse roles in different species. In addition, a group of cys-loop LGIC subunits that appear to be particular to insects, which was noted as *D. melanogaster *Group 1 [62], and denoted here as Insect Group 1 to accommodate sequences from other species (Fig. 5), may represent more recent members of the superfamily since only one subunit is found in *Apis *and three are present after the emergence of Coleoptera. It will be of interest to determine the functional role played by Insect Group 1 subunits and the ligands to which they respond.

The species-specific diversification arising from alternative splicing, RNA editing and divergent subunits, as well as insect-specific subunits, represents promising receptor differences to target for the future rational design of insecticides that control pest species while sparing beneficial insects. The use of heterologous expression systems such as *Xenopus laevis *oocytes has allowed the functional characterisation of several *Drosophila *cys-loop LGICs such as RDL [56], GRD and LCCH3 [55], GluCl [67], HisCl1 and HisCl2 [5,6] and pHCl [59]. Similar studies of heterologously expressed ion channels from other insect species including *T. castaneum*, in combination with the use of three-dimensional models based on the crystal structure of molluscan acetylcholine binding proteins [1,68-70], will likely prove useful in the search for novel compounds that show selectivity for receptors of certain insect species as well as in determining the mechanisms of insecticide interaction with cys-loop LGICs. For insect nAChRs, functional expression in heterologous systems has so far proven elusive [1], although low levels of receptor activity have been observed for the locust *Schistocerca gregaria *αL1 subunit expressed in *Xenopus *oocytes [71]. Nevertheless, *Drosophila *nAChR α subunits can form robust functional channels when coexpressed with a vertebrate β2 subunit [72] and studies on such hybrid receptors have provided insights into the selectivity of neonicotinoids for insect nAChRs over those of vertebrates, regions of subunit proteins involved in imidacloprid interactions and the actions of different neonicotinoids [73]. These studies have highlighted Dα1 and Dα2 as being sensitive to imidacloprid. Also, Mpα2, which is the aphid *Myzus persicae *ortholog of Dα1, shows high levels of imidacloprid binding when coexpressed with the rat β2 subunit [74]. The functional expression of nAChRs with insect β subunits has yet to be achieved but it is worth noting that the ortholog of Dβ2 is an α subunit (e.g. Tcasα8 in *Tribolium*) in all insects so far studied outside the *Drosophila *genus [36]. Since members of the Dβ2 group are closely related to those of the Dα1 and Dα2 groups (Fig. 5) and share an insertion in loop F which may contribute to imidacloprid sensitivity [38], it would be of interest to determine whether Tcasα8 is sensitive to neonicotinoids. If this is the case, it would also be worth studying Tcasα11, which appears to be a product of gene duplication of Tcasα8, and assessing how both subunits contribute to neonicotinoid sensitivity particularly in light of the finding that gene duplication has given rise to insecticide resistance at another synaptic target site, acetylcholinesterase [75].

Parental RNAi, where RNA interference arising from double-stranded RNA introduced into the mother also spreads to the offspring, is highly efficient in *Tribolium *[27,76]. The combination of genome information and the use of RNAi in *C. elegans *has proved considerably instructive in determining roles played by genes [77,78]. Thus, the beetle provides a powerful tool for studying gene function in an insect pest species. For example, RNAi could be used to elucidate the roles played by *Tribolium *cys-loop LGIC subunits in various aspects of development, behaviour and response to insecticides. Recently, it has been shown that a *Drosophila *Dα6 knockout mutant is highly resistant to spinosad [79]. Studies have shown that *T. castaneum *is susceptible to spinosad, although to a lesser degree than other insect pests of stored wheat [80]. It will be of interest to determine the effect of silencing Tcasα6 on the beetle's susceptibility to spinosad and perhaps validate in a pest species findings based on the *Drosophila *genetic model organism.

## Conclusion

Using information from the *Tribolium castaneum *genome sequencing project, we report, for the first time from an invertebrate pest species, a complete cys-loop LGIC superfamily, which encodes for receptors that play important roles in the nervous system as well as for targets of widely-used insecticides. The present study enhances our understanding of the functional genomics of the insect cys-loop LGIC superfamily. Our findings reveal an emerging consensus that in over 300 million years of insect evolution, the cys-loop LGIC superfamily has remained compact with only minor changes in gene numbers. However, alternative splicing, RNA editing and the presence of divergent subunits broadens the cys-loop LGIC proteome and generates species-specific receptor isoforms. Thus, the paper provides several new insights into the molecular diversity of cys-loop LGICs between different organisms and provides an important foundation for associating particular cys-loop LGIC subtypes with development as well as for the generation of improved insecticides that target the red flour beetle.

## Methods

### Identification of cys-loop LGIC subunits in the *T. castaneum *genome

To identify putative cys-loop LGIC subunits, we screened the *T. castaneum *genome (assembly version 2.0) [81] with cDNA sequences of every member of the *D. melanogaster *and *A. mellifera *cys-loop LGIC superfamilies using the tBLASTn algorithm [28]. Candidate beetle cys-LGIC subunits were identified based on their considerable sequence homology with previously characterized subunits (sequences with lowest similarity had E Value 8e-19), particularly in the N-terminal ligand-binding domain and the four transmembrane regions. The highly variable N-terminal signal peptides, which are a feature of cys-loop LGIC subunits, were identified in the GLEAN consensus set of predicted genes [27]. RT-PCRs were performed [see Additional file 1 for primers used] to verify and correct the open-reading frame sequences of each subunit.

### Reverse transcription and polymerase chain reaction

Total RNA was extracted from 15 *Tribolium castaneum *adult beetles (Georgia GA2 strain) homogenized in Trizol (Invitrogen) using the RNeasy Mini Kit (Qiagen) and first strand cDNA was synthesized from 1 μg total RNA using Superscript™ III First-Strand Synthesis Super Mix (Invitrogen). Nested RT-PCR reactions were performed to detect transcripts of beetle cys-loop LGIC subunits as well as to detect transcript variants arising from alternative splicing. Primer pairs [see Additional files 1 and 3 for primer sequences] recognising different exons were used to allow identification of cDNA-specific products. The PCR reactions were performed in a total volume of 50 μl composed of *Taq *polymerase and 1 × PCR buffer (Sigma), 0.2 mM dNTP mix (Roche), 0.4 μM each primer and 2 μl first strand cDNA template. The PCR reaction conditions were 35 cycles of: 95°C for 30 s, 55°C for 30 s, 72°C for 90 s. The first PCR was used at a final dilution of 1 in 5000 as template for the second, nested PCR reaction. For Tcas_RDL and Tcas_GluCl, a 1 in 500 dilution was used to amplify enough DNA for sequencing. DNA sequence chromatograms for each cys-loop LGIC subunit were analyzed using Chromas 2 (Technelysium Pty Ltd) to detect single nucleotide polymorphisms (SNPs) or RNA editing sites as shown by mixed signal peaks. No SNPs were observed and the putative RNA editing sites detected in Tcasα6 and Tcasβ1 were verified by amplifying and sequencing genomic DNA present in the extracted total RNA, which was first treated with DNase-free RNase (Roche), using primers recognising intron DNA [see Additional file 4 for primers used]. Sequence chromatograms showing a defined region of mixed peaks indicated differential splicing. The corresponding cys-loop LGIC PCR products were cloned into the pGEM-T Easy vector (Promega) and between 10 to 20 transformants were sequenced to identify individual subunit isoforms. All PCR products were analyzed by electrophoresis in a TAE gel and then purified using the QIAquick Gel Extraction Kit (Qiagen) while subunits cloned in pGEM-T Easy were purified using the QIAprep Spin Miniprep Kit (Qiagen). Purified DNA was sequenced by the dye termination method at Cogenics [82]. For analyzing RNA editing levels in Tcasα6 splice variants, RT-PCR was performed using forward primers specific to either exon 3a or exon 3b and reverse primers recognising one of the three alternatives for exon 8 [see Additional file 3 for primers used]. A nested PCR approach was adopted since two rounds of PCR were required to amplify enough variant containing exon 8c for visualization on an agarose gel. The proofreading Pfu Turbo DNA polymerase (Stratagene) was used in 2 × 30-cycle reactions on four independently made first-strand cDNAs. The sequence chromatograms of the amplification products were analyzed to give editing levels where the proportion edited = height of guanosine peak/(height of guanosine peak + height of adenosine peak). One-way and two-way ANOVA analyses were performed with Turkey's Multiple Comparison and Bonferroni tests respectively using Graphpad Prism 4 [83]. Products amplified by either one or two PCR reactions gave similar editing levels.

### Sequence analysis

The multiple protein sequence alignment was constructed with ClustalX [84] using the slow-accurate mode with a gap opening penalty of 10 and a gap extension penalty of 0.1 as well as applying the Gonnet 250 protein weight matrix [85]. The protein alignment was viewed using GeneDoc [86]. Identity values between subunit sequences were calculated using the GeneDoc program. The neighbour-joining method [87] and bootstrap resampling [88], available with the ClustalX program, were used to construct a phylogenetic tree, which was then displayed using the TreeView application [89]. Signal peptide cleavage sites were predicted using the SignalP 3.0 server [90] and membrane-spanning regions were predicted using the TMpred program [91]. The PROSITE database [92] was used to identify potential phosphorylation sites.

## Abbreviations

ACh – acetylcholine, CLGC – cys-loop ligand-gated ion channel, GABA – γ-amino butyric acid, GluCl – glutamate-gated chloride channel, GRD – GABA_A _and glycine receptor-like subunit of *Drosophila*, HisCl – histamine-gated chloride channel, LCCH3 – ligand-gated chloride channel homolog 3, LGIC – ligand-gated ion channel, nAChR – nicotinic acetylcholine receptor, RDL – resistant to Dieldrin, RNAi – RNA interference, TM – transmembrane domain

## Competing interests

The author declares there are no competing interests.

## Authors' contributions

AKJ carried out all the work presented in the study and drafted the manuscript. DBS participated in the design of the study and was involved in critically revising the manuscript for important intellectual content. All authors read and approved the final manuscript.

## Supplementary Material

Additional file 1 Sequences of primers used to amplify open reading frames of *T. castaneum *cys-loop LGIC subunits. The table provided shows the oligonucleotide DNA sequences used in PCR to amplify open reading frames of *T. castaneum *cys-loop LGIC subunits. Click here for file

Additional file 2 Protein sequence alignment of *T. castaneum *cys-loop LGIC subunits. The figure provided compares protein sequences of *T. castaneum *cys-loop LGIC subunits with each other as well as with Drosophila Dα1 and RDL. Click here for file

Additional file 3 Sequences of primers used to amplify alternate splice variants of *T. castaneum *cys-loop LGIC subunits. The table provided shows the oligonucleotide DNA sequences used in PCR to amplify alternate splice variants of *T. castaneum *cys-loop LGIC subunits. Click here for file

Additional file 4 Sequences of primers recognising intron DNA of *T. castaneum *cys-loop LGIC subunit genes. The table provided shows the oligonucleotide DNA sequences used in PCR to amplify genomic DNA of *T. castaneum *cys-loop LGIC subunits. Click here for file
