# Supplementary material for: The cys-loop ligand-gated ion channel gene superfamily of the red flour beetle, Tribolium castaneum
Source: BMC Genomics. 2007 Sep 19;8:327. doi: 10.1186/1471-2164-8-327 (PMC2064938; doi:10.1186/1471-2164-8-327)
Supplement: Additional file 1 — Sequences of primers used to amplify open reading frames of T. castaneum cys-loop LGIC subunits. The table provided shows the oligonucleotide DNA sequences used in PCR to amplify open reading frames of T. castaneum cys-loop LGIC subunits. [file 1471-2164-8-327-S1.pdf]

Supplemental Material Table S1. Sequences of primers used to amplify open reading frames of *T. castaneum* cys-loop LGIC subunits. Forward primers are on the top of each pair and reverse primers are on the bottom.

Subunit	1st PCR reaction	2nd PCR reaction
Tcasα1	atggagctgttgctcgcc +	cctttcatggttgattttgg +
	ttacaactcctccggtac	tcaacaacgccattttcttc
Tcasα2	atgatgtggtggtggctg +	ttcggactgtttttgtcgg +
	ttagaaatctaattcaggtaga	gttgttgggctactgacg
Tcasα3	atgaagagcttggtgggg +	tgtggatagttttagtgctc +
	taaagaactggcaatatggg	gcagtggtcgatgcaatc
Tcasα4	atgcctccttctgccgc +	gagacgctgagagcctg +
	ctactgtggaggagggc	gaacaaccgtagtggtgg
Tcasα5	ttgatagttgtaatcctgttg +	gagaatgtcaaggggacg +
	agaggccgcgtcgtttg	gacttctgcataatatcgtc
Tcasα6	atggtccggagcgagca +	tggcgctgctggccac +
	ctactgcacgattatgtgc	gtggcgatgatcgtgaac
Tcasα7	atggacagtggcatgcag +	tgtcaattctactgcggtc +
	acgatacgatgatgtgagg	ccgagaacagcactgcta
Tcasα8	tttaataaaattagtttgtttcac +	catgctgtttaaatccactg +
	gtggggatgtgtgtgcg	ttcgtgagaggaatttcgc
Tcasα9	atgggcattctaaattattttc +	cctttcagctgcgtcttg +
	taaggggtgaacccgataa	aacaatcacgtagacgagg
Tcasα10	atggcaaacatactgaggc +	tggtgtgcggcttttgca +
	tcacacggaataaactatgg	aaagtgatccgatctatggc
Tcasα11	tgtacttgttaaagtttacatg +	gtttaagtttgttgttggttg +
	tacaacctcaaagcgccc	atctactggaatcctcgtg
Tcasβ1	atgtggacctggaccgc +	acagcggtcttcatctcg +
	tatttgccgcggtaaatttc	attcgaagatgtgaggagc
Tcas_RDL	gggtggtgtggccagc +	gtgctgctggccctcg +
	acttatcctcctccagaag	tagatcgtccgctacaaca
Tcas_GRD	gaagcggtttcaactttgg +	gaccgtttcgctgctaac +
	tctcttggtcctcgacag	tactctagaagctcgatcaa
Tcas_LCCH3	atgatcacgattatctacatc +	catttccattgaggtatattc +
	aacagccagtacccagca	agtaaccgggaaaataatcc
Tcas_GluCl	gtatacacacaccctcatc +	gatagtgcacatcatccac +
	ttacgactcttcggcctc	tatattacattgaagagtgcg
Tcas_HisCl1	gcctccgttttcgttagg +	tgggtggttccgattacc +
	tactcagcgaacattatcca	aggacagtgaagagcaaag
Tcas_HisCl2	gtattctgtgttttactggc +	ctgtgtatgatctcaaatgta +
	aacgtcgtccagtacacaa	actggtcaatacagagagc

Tcas_pHCl	atgggtcagccttggatg + ttgtgagtcagagtgagtg	ggctgctatacttctagtg + aactgctgtagtgatagaag
Tcas_8916	ttcgggccggtttttgtc + cagtgactaagcgagctc	ttgttcggaagctttgagg + cttcgtatccgcccattta
Tcas_12344	tgtgggttgtggtgacag + acaacaatgtcgcacttcta	aagtacgacctcctactca + gtccagtaaatcgcacaga
Tcas_6927	atgcagaaactcgttctc + acaacatagacgaaagacca	atgtgcacaagggcccag + tccagttggcgacctcg
Tcas_7589	tgctaaagtggcggcttg + gcgggaacacgattcttg	ttgcctcgtttatgactgg + tttcctgtcgatccagttg
Tcas_11340	gactagtgcagttctagtc + tatacaaacacccagtagag	agtgattgaaggagaagcc + tgagtgggaagacaattcg
