# Supplementary material for: The cys-loop ligand-gated ion channel gene superfamily of the red flour beetle, Tribolium castaneum
Source: BMC Genomics. 2007 Sep 19;8:327. doi: 10.1186/1471-2164-8-327 (PMC2064938; doi:10.1186/1471-2164-8-327)
Supplement: Additional file 3 — Sequences of primers used to amplify alternate splice variants of T. castaneum cys-loop LGIC subunits. The table provided shows the oligonucleotide DNA sequences used in PCR to amplify alternate splice variants of T. castaneum cys-loop LGIC subunits. [file 1471-2164-8-327-S3.pdf]

Additional file 3. Sequences of primers used to amplify alternate splice variants of *T. castaneum* cys-loop LGIC subunits. Forward primers are on the top of each pair and reverse primers are on the bottom. All primers are shown 5' to 3'.

Subunit Variant	1st PCR reaction	2nd PCR reaction
Tcasα6 3a8a	tggcgctgctggccac + gcagttgaaataggtccc	gaaaagaatcagattctcacg + gaactgcatcggacgtgg
Tcasα6 3a8b	tggcgctgctggccac + gcagttgaaataggtccc	gaaaagaatcagattctcacg + aaggaatggcgtcagatac
Tcasα6 3a8c	tggcgctgctggccac + gcagttgaaataggtccc	gaaaagaatcagattctcacg + gacgtctgggttatcacg
Tcasα6 3b8a	tggcgctgctggccac + gcagttgaaataggtccc	ggacgagaagaatcaactc + gaactgcatcggacgtgg
Tcasα6 3b8b	tggcgctgctggccac + gcagttgaaataggtccc	ggacgagaagaatcaactc + aaggaatggcgtcagatac
Tcasα6 3b8c	tggcgctgctggccac + gcagttgaaataggtccc	ggacgagaagaatcaactc + gacgtctgggttatcacg
Tcas_RDL 3a6a	gtgctgctggccctcg + tcccaaatagacgtcgatc	ctcagtatcagctccgtc + ggtcaggttaattacggtc
Tcas_RDL 3a6b	gtgctgctggccctcg + tcccaaatagacgtcgatc	ctcagtatcagctccgtc + gtgaggctgatctccatg
Tcas_RDL 3b6a	gtgctgctggccctcg + tcccaaatagacgtcgatc	ctcgctgtccgaagtgaa + ggtcaggttaattacggtc
Tcas_RDL 3b6b	gtgctgctggccctcg + tcccaaatagacgtcgatc	ctcgctgtccgaagtgaa + gtgaggctgatctccatg
Tcas_RDL 3c6a	gtgctgctggccctcg + tcccaaatagacgtcgatc	tcgctgtccgaggtcca + ggtcaggttaattacggtc
Tcas_RDL 3c6b	gtgctgctggccctcg + tcccaaatagacgtcgatc	tcgctgtccgaggtcca + gtgaggctgatctccatg
