# Supplementary material for: The cys-loop ligand-gated ion channel gene superfamily of the red flour beetle, Tribolium castaneum
Source: BMC Genomics. 2007 Sep 19;8:327. doi: 10.1186/1471-2164-8-327 (PMC2064938; doi:10.1186/1471-2164-8-327)
Supplement: Additional file 4 — Sequences of primers recognising intron DNA of T. castaneum cys-loop LGIC subunit genes. The table provided shows the oligonucleotide DNA sequences used in PCR to amplify genomic DNA of T. castaneum cys-loop LGIC subunits. [file 1471-2164-8-327-S4.pdf]

Additional file 4. Sequences of primers recognising intron DNA of *T. castaneum* cys-loop LGIC subunit genes. Forward primers are on the top of each pair and reverse primers are on the bottom. All primers are shown 5' to 3'.

Subunit	1st PCR reaction	2nd PCR reaction
Tcasα6	tctatcggtctggtgacg +<br>tgcagtgtgggaagtgtg	tggtctcacggtctctga +<br>gcgtgtgatgtggtagca
Tcasβ1	ttcgagacctcttccgag +<br>gttccctttttgccaaatgg	gtacaacaagctgatacgc +<br>gggtaacctgagtacagc
